# Food safety risk‐assessment systems utilized by China, Australia/New Zealand, Canada, and the United States

**DOI:** 10.1111/1750-3841.16334

**Published:** 2022-10-26

**Authors:** Sieh Ng, Shuyan Shao, Nan Ling

**Affiliations:** ^1^ CSIRO, Agriculture and Food Werribee Victoria 3030 Australia; ^2^ Nanjing Weigang Dairy Co., Ltd Nanjing Jiangsu Province China

**Keywords:** food fraud, food safety, risk assessment

## Abstract

Ensuring the chemical, physical, and microbial safety of food and ingredients underpins the international trade of food items and is integral to building consumer confidence. Achieving this requires effective systems to support the safety of food across the supply chain. Differing risk‐assessment approaches are utilized globally for establishing food safety systems, and bench marking these approaches against international food safety standards can assist in the development of country‐specific systems. This China–Australia collaborative review examined similarities and differences in the food safety risk‐assessment systems of China, Australia/New Zealand, Canada, and the United States, with the view to identify areas that could support improvements to the Chinese system. Key differences include the level of cohesiveness among stakeholders and the level to which each country promotes the international harmonization of standards. The evidence highlights a need for greater capacity‐building in risk assessment in China that may support greater stakeholders’ cohesion, improve hazard identification, and allow regulators to more readily keep abreast of changes to international standards. This review may help the Chinese food industry to replicate the same level of food safety risk assessment currently applied by other key countries, and reflects the determination, government prioritization, and active strengthening of China's National Centre for Food Safety Risk Assessment currently underway.

## 1 INTRODUCTION

### 1.1 Food safety risk assessment at the international level

The extent of global international trade among countries invariably leads to different countries evaluating food safety standards with reference to the international food safety standards, the Codex Alimentarius Commission (Codex) and disputes pertaining to food safety standards are referred to the World Trade Organisation (WTO) (Jackson & Jansen, 2010). In the food safety risk‐assessment process, scientific evidence on food safety is collected and is used to aid in the setting of international food standards by Codex, whereas trade disagreements are addressed by WTO. There is an agreement between Codex and WTO on the Application of Sanitary and Phytosanitary Measures in which WTO declares that Codex is the reference for international food standards. Risk assessments for food safety provide information that leads to risk management. The relationship between the two is complex. Based on information from risk assessments, risk management determines which policies to adopt in order to reduce consumer risk. Risk managers regularly have to consult with risk assessors who have knowledge that can enhance their management of risks.

In terms of food safety risk assessment, it is difficult to maintain as much neutrality as possible with regard to internationally recognized standards, since many parties have a vested interest, such as exporting and importing countries.  Exporting countries would adhere to the Codex standard for food safety, while importing countries may try to protect their local manufacturers' interests by imposing more stringent requirements on importing products, including non‐monetary trade tariffs and sanitary and phytosanitary requirements. The importing countries would have to provide scientific proof that the more stringent food safety requirements are justified for the intended use of the product in the importing country and for the specific consumer sector. Risk assessments for food safety provide information that leads to risk management. A risk manager's responsibilities also include providing feedback and contributing to the risk‐assessment process, making these two functions somewhat inseparable and interconnected; hence, the risk‐assessment process may not be as unbiased as Codex intended (Jackson & Jansen, 2010).

Global international trade is happening more frequently than ever, and this comparison of the food safety risk‐assessment systems employed in China, Australia, New Zealand, the United States, and Canada highlights the differences in food safety standards often applied to products destined for exports and imports. The exporting countries would generally like to impose less stringent food safety requirements on their products so that it can enable the increase in sales of their export markets, whereas the government authority of importing countries generally imposes a more stringent food safety requirement on imported goods in an attempt to protect the local industry and their population. In a situation where the food safety standards deviate from the Codex Alimentarius Standards, the importing countries would need to provide scientific evidence to justify a more stringent food safety standard requirement. This can create conflict in the trade, leading to the World Trade Organisation having to deal with disputes in food safety standards used by exporting and importing countries.

Considering that China exports a large amount of food to the world and deals with many different international markets, China and other exporters may improve food safety involved in business transactions with international markets by reviewing the risk assessments and food safety standards of the importer countries. China exports and imports a lot of products to Australia, New Zealand, the United States, and Canada, hence this study was initiated to improve understanding of how various countries’ food safety systems work.

### 1.2 Risk analysis in food regulation

The purpose of risk analysis in food regulation is to maintain a safe food supply. Food safety responsibility lies with the whole food supply chain, from “farm‐to‐fork.” It involves risk assessment and risk management of all the food safety hazards including the microbiological, chemical, and physical hazards starting from the raw material supply, through food processing into retail where processed food is sold, food service where food is delivered to consumers, and in the kitchens of consumers where proper food handling needs to be implemented to avoid contamination. Therefore, ensuring the safety of food involves many different stakeholders including producers and growers, food manufacturers, retail, and food service. It is also the responsibility of the government to ensure the implementation of a safe food system and for consumers to store, use, and prepare food as intended in a hygienic way to limit the chances of contamination, which would jeopardize the integrity of the food, rendering it unsafe (FSANZ, 2013a).

The collaborative efforts of government, food industry, and consumers are key to ensuring that the safety of food is achieved. Food supply chains are constantly changing due to new innovative technologies, which occur in response to consumer demands for fresher, tastier, better color retention, more nutritious, and extended shelf‐life products. Free trade agreements between international trade partners, emergence of migrants from international backgrounds bringing in various ethnic preferences, and changing diets from increased consumer interest for plant‐based products, novel proteins, and fermented foods like kimchi, sauerkraut, tempeh and kombucha, are experiencing renewed or broader interest (Landscape news, 2020). The dynamic plant‐based protein market is gaining momentum with a growing number of consumers who care about global sustainability and animal welfare. As a result, the size of the international plant‐based protein market is predicted to increase from US$10.3 billion in 2020 to US$14.5 billion by 2025 (Askew, 2021). Food trends are moving from healthy innovation to personalized nutrition (Archer et al., 2017; Betts & Gonzalez, 2016) with functional foods an emerging area. Moreover, it is predicated that the next generation of 3D printing technologies will emerge as the next food trend (Chadwick, 2017), with the ability to create esthetically pleasing and intricately shaped foods, that are more complex in nature, nutritionally balanced, and optimized for texture and taste (Watkins et al., 2022). With all these changes, food safety has never been more important and needs to be carefully considered when new product development and new innovative food technologies are introduced (Buckow et al., 2013; Juliano et al., 2021; Knoerzer & Muthukumarappan, 2021; Knoerzer et al., 2011).

Various international committees have been established to help provide guidance around risk‐based systems for various foodborne hazards. These include the Joint FAO/WHO Expert Committee on Food Additives (JECFA), which provides chemical risk studies on additives in food, and the Joint FAO/WHO Expert Meetings on Microbiological Risk Assessment (JEMRA), which provides risk management advice on microbiological food safety issues. Both provide important insights and guidance for the preparation of food safety systems that are based on risk calculation.

### 1.3 Risk management

Microbiological risk management is a complex issue, encompassing analysis of epidemiological data, pathogenicity of microorganisms of concern, a good knowledge of the product formulation to determine how it can support microbial growth, and the preventive measures that can be administered in food. “Food Safety Objectives” (FSO) is a very useful microbiological risk‐management tool that provides information that enables a food processor to implement a sufficient microbial reduction step in processing so that the microbiological hazard of concern in a product at the point of consumption does not exceed the allowable limit. The use of FSO is recommended by the International Commission on Microbiological Specifications for Food (ICMSF, 2011).

### 1.4 The development of food law and regulation

#### 1.4.1 The evolution of food‐safety establishments in China

In 2003, the government took the decision of closing down a substantial number of small family businesses on the ground of discovering unsafe food manufacturing practice that jeopardized the production of a safe food (Tam & Yang, 2005). With the rise of an era of an affluent middle‐class society in China from around 2005 (Yuan et al., 2011), China's regulatory agency has focused particular attention on its consumer safety due to an increase in demand by consumers that have better food safety knowledge and expect value for their money. Previously, there was an un‐unified and unclear authority among the various regulatory administrations which in turn presented a notable issue for food safety regulation. The reform in government at that time gave rise to a temporary measure and created a void in the regulatory system (Tam & Yang, 2005). The need for government to generate employment had created a conflict on the implementation of food safety regulation by allowing food handlers without food safety knowledge to operate in the food processing area.

The food regulatory system of China has continued to develop over time, and consists of a three‐layered system based on basic, subordinate, and enforcement laws. The major development of the basic laws is that it enjoys the highest hierarchy status compared with all other laws. This is followed by the subordinate laws, which have jurisdiction over food businesses, ensuring the labeling of foods conform to regulatory guidelines and clamping down on harmful additives in food. The subordinate laws differ in function compared to the basic laws but complement the basic laws by issuing guidelines in ensuring processed foods are safe for human consumption. The whole food safety system functions efficiently with the aid of the relevant provincial government in setting control specifications. The government of China established the Food Safety Law (FSL) in 2009, which was developed from the pre‐existing Agrifood Quality and Safety Law implemented in 2006, and before that, the Agriculture Law in 2003 (Zhang et al., 2018). The FSL was a pioneer in breaking through the long history of a convoluted food safety system, and ensures the China food safety system upholds the safety and quality of food, and hence public health. The FSL also manages food safety incidents by providing rectifying recommendations.

The basic laws are responsible for the quality and safety of agricultural food, and their administration falls under the jurisdiction of the Ministry of Agriculture. Its function is to ensure compliance with the fresh produce cultivation, farming practices, fresh produce processing in the factory, food packaged in hygienic conditions, and all other safe food practices in a processing plant (Wang, 2010).

Food safety has become a high priority and focus for the Chinese government and has motivated a transformation of the food safety legislative framework resulting in the establishment of China's National Centre for Food Safety Risk Assessment (CFSA, https://en.cfsa.net.cn). CFSA is hailed as a revolution to food safety bench marking and setting of national standards and led to the creation of the National Food Safety Standards (NFSS). These standards were largely based on the Codex Alimentarius Commission (CAC) and, as such, the best practices of developed countries. The aligned risk‐assessment protocols currently employed in China are underpinned by a comprehensive research dataset of relevance to China in relation to food production and consumer practices (Zhang et al., 2018). The Agrifood Quality and Safety Law that was established in 2006 ensures agricultural product's quality and safety are maintained, reducing public health issues related to agricultural products and advances the economy related to agriculture. The Agriculture Law that was formed in 2003 strengthened the value of agriculture, improved the national economy, promoted changes in rural areas, and increased the productivity of the agriculture sector (Agriculture Law of the People's Republic of China, 2003).

#### 1.4.2 The Australia and New Zealand food regulatory system

The Australia and New Zealand food regulatory system consists of three major elements including the development of food policy, establishment of food standards, and overseeing the implementation of these food standards and policy. This system is multifaceted and involves various sectors which include the Australian and New Zealand governments, supplemented by governmental enforcement agencies from the states and territories of Australia and local municipal councils. The Food Minister's Meeting (previously the Australian and New Zealand Ministerial Forum on Food Regulation) is responsible for policy development and is supported by the Food Regulation Standing Committee. In Australia, the administration of food safety is a cooperative task that is shared by both central and state governments. Ensuring the safety of imported foods falls under the jurisdiction of the Department of Agriculture, Water and the Environment (DAWE), who oversees inspection of imported and exported food. In New Zealand, it is the Ministry for Primary Industries that governs the inspection of imported and exported food.

#### 1.4.3 USA food safety system

In the USA, the US Food and Drug Administration (FDA) employs a strategy that is risk‐based for food safety management, and this strategy consists of nine steps, as shown in Table 1. The food safety system of the United States comprises the federal regulatory system and the state regulatory program (U. S. Food and Drug Administration, 2021). For the federal entities, the major agencies are the Food and Drug Administration (FDA), Food Safety and Inspection Service, Environmental Protection Agency (EPA), and the National Marine Fisheries Service (NMFS). The FDA has jurisdictional authority over local and imported foods, excluding meat and poultry. The Center for Food Safety and Applied Nutrition (CFSAN) within FDA oversees the safety, nutritional values, sanitary conditions, and whether products are correctly labeled within the food sector. The EPA, on the other hand, is responsible for the licensing of all pesticide products, whereas the NMFS provides the assessment and grading of seafood. There are in excess of 3000 state and local agencies assisting the FDA in regulating the food safety practice for the hospitality, food service, and retail sectors (Ensuring Safe Food, 1998).

**TABLE 1:** USFDA food safety management strategy

Step No.	Food safety management
1	Plan strategically, consultation with various stakeholders to identify food safety objective
2	Rank the risk associated with public health
3	Gather specific information related to the risk studied
4	Evaluate to score the risks with regards to impact on public health
5	Consult with other major stakeholders in food safety
6	Evaluate factors including consumer concerns, market impact, detrimental environment impacts
7	Prioritizng manpower to manage the risks
8	Continuous assessment of the efficiency of the risk‐management system
9	Conducting all duties in a coordinated and unbiased manner involving all relevant stakeholders

*Source*: Table information adapted from Wallace and Oria (2010).

#### 1.4.4 Canada food safety system

In Canada, the food safety regulatory bodies are Health Canada (HC) and the Canadian Food Inspection Agency (CFIA). HC has jurisdiction over the health protection rule on a federal basis and regulates the food safety measures of the Food and Drugs Acts (Health Canada, 2021). It relies on various Acts that were developed to address food safety risks associated with health of the public and the CFIA is the enforcement body to ensure compliance with the Acts (CFIA, 2021). Other supporting agencies include the Agriculture and Agri‐Food Canada and the Public Health Agency of Canada.

## 2 THE COMPARISON OF THE FOOD SAFETY RISK‐ASSESSMENT SYSTEMS AMONG CHINA, AUSTRALIA, NEW ZEALAND, THE UNITED STATES, AND CANADA

### 2.1 Risk‐assessment methodologies

**FIGURE 1:**
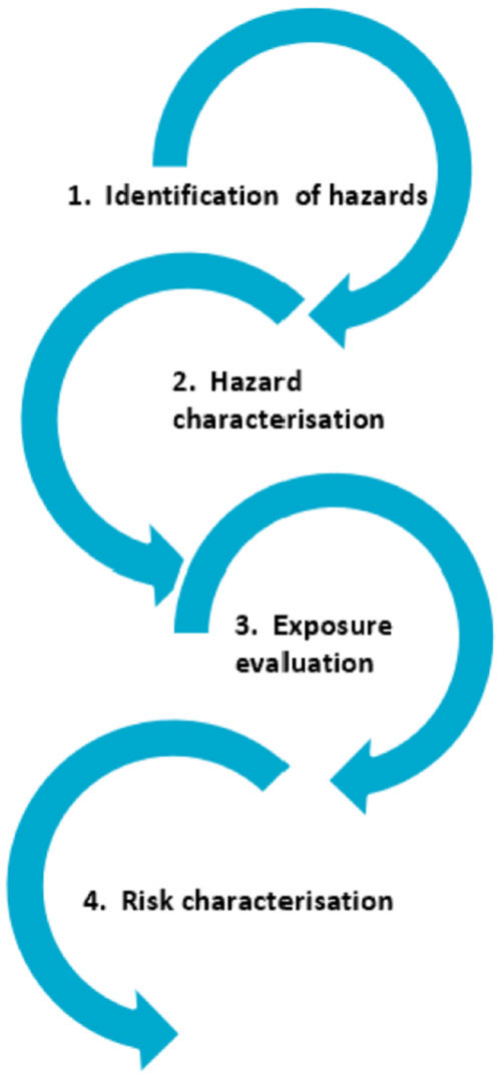
Food Safety Risk Assessment system (adapted from FSANZ, the four key steps in risk assessment, 2013b)

Food safety risk‐assessment systems of China, Australia/New Zealand, the United States, and Canada utilize the Codex Alimentarius food safety risks assessment system. Figure 1 shows the basic process of food safety risk assessment per the Codex Alimentarius system, and how it has been applied by Australia and New Zealand. Step 1 and 2 involves the identification and characterization of hazards which, when present in foods, can cause adverse health effects. Hazard characterization involves an evaluation of the qualitative or quantitative nature of the adverse health effects. Step 3 includes the application of what is referred to as an “exposure assessment,” which aims to understand the likely intake of hazards through consumption of foods (Fischer et al., 2005) and step 4 provides an estimation of the likelihood that a hazard could occur (FSANZ, 2013b). Despite the similar approaches taken by each of the different countries for their food safety risk‐assessment systems, there are several key differences between China (Li & Liu, 2017; Wu & Chen, 2013; Wu et al., 2018; GB 2763‐2019, 2019), Australia/New Zealand (AGAPVMA, 2020; Amis, 2020; FSANZ, 2013c; ICMSF, 2011), Canada (Bietlot & Kolakowski, 2012; CFIA, 2021; Health Canada, 2021), and the United States (FDA iRISK, 2021; FoodRisk.org, 2017; JECFA, 2021; USDA, 2021), which are described in detail in the sections below.

Australia/New Zealand, Canada, and the United States have been applying risk‐assessment methodologies following international standards such as FAO/WHO Codex Alimentarius Commission over the past 30 years. This ensures that food safety risk assessments are based on the latest scientific evidence and recently published databases and reflect extensive national and international findings on food safety risks and proven control measures to manage the risks and ensure a safe food supply for consumers. More recently, China embarked on using the FAO/WHO Codex Alimentarius Commission risk assessment tool after a reform in the nation's food safety regulation in 2009, where China launched the Food Safety Law. Despite China making advances with its risk‐assessment methodologies and accumulating scientific databases over the past decade, an undeniable gap still exists between China and countries more advanced in their food safety risk assessment (Li & Liu, 2017). To address this, China needs to work closely with international food safety agencies to improve its risk‐assessment methodologies.

The risk‐assessment methodology used by the CFIA in Canada is a scoring system known as the “Ranked Risk Assessment” and used to score chemical hazards according to their “relative risk.” The relative risk for a chemical present in a food is estimated by calculating the toxicity of that chemical and the likelihood. In Canada, the “risk model scoring system” works hand in hand with the Risk Priority Compound List (RPCL) (Bietlot & Kolakowski, 2012). The RPCL consists of various categories of toxic chemicals present in humans and animals. Category A caters to toxicity for the acute and chronic animal toxicity; Category B focuses on impact on human health; and Category C covers foods of plant and animal origin. The relative ranking is calculated by the information created by the monitoring and analysis programs.

In the United States, the US FDA is the government department that oversees food safety risk analysis, risk assessment, risk management, and risk communication. Within the FDA, there is the specialized department known as the Centre for Food Safety and Applied Nutrition (CFSAN), which undertakes risk analysis to develop the best solutions. The FDA uses a risk analysis program and a set of tools available on the web‐based platform, foodrisk.org (FDA iRISK, 2021). Under the umbrella of the food risk analysis program is the Joint Institute of Food Safety and Applied Nutrition (JIFSAN). JIFSAN's strategy focuses on making use of sound scientific findings to promote collaborative research and it is heavily involved in sustainable partnerships with research organizations, academia, and global collaborators in food safety risk assessment. There is also a strong emphasis on the importance for the provision of training by government to cultivate skilled labor in the food sectors and building capacity in the risk analysis of food safety through improving education. Other tools provided by FoodRisk.org include the Interactive online Catalogue on Risk Assessment (ICRA), the Food Commodity Intake Database (FCID), the Produce Point of Origin Database (PPOD), the Norovirus database (NoroDB), and the Violations Database, which specifically focuses on food safety inspections for retail entities.

### 2.2 Monitoring and enforcing chemical residue and contaminant limits in food

In Australia, all chemicals used in the agriculture field and veterinary sectors are governed by the Australian Pesticides and Veterinary Medicines Authority (APVMA), who establishes the maximum residue limits (MRLs) for chemicals. The protocol of setting limits for all chemicals is based on scientific findings through research and clinical trials provided by research organizations and academic institutions. As long as the chemicals are used by industry in accordance with the manufacturer's instruction on the label, there is an assurance that the chemical will be administered safely, so that MRLs are not exceeded. Chemical contaminants that can infiltrate through diet are also being evaluated by APVMA (Australian Government Australian Pesticides and Veterinary Medicines Authority, 2020). To ensure MRLs are valid, national and international scientific methods are utilized by FSANZ and APVMA. FSANZ also recognizes the value of harmonization MRLs with limits at the same standard with Codex Alimentarius. In Australia, the state regulatory bodies are the agencies that enforce adherence to the MRLs in food. APVMA works closely with FSANZ to compile the information for the new registration of chemicals (FSANZ, 2013a). In New Zealand, it is the Ministry of Primary Industries that enforces the MRLs in food (Ministry for Primary Industries, 2021).

In China, the process of setting the limits of pesticide MRLs is based on a residual chemical assessment, which includes agriculture sector experiments, food manufacturing, climatic/environmental factors, toxicology study, and an evaluation of dietary intake (Chen et al., 2015). China's National Health Commission, the State Administration for Market Regulation, and the Ministry of Agriculture and Rural Affairs co‐govern the MRLs for Pesticides in Foods, according to the Chinese national standard, GB, 2763‐2021 (GB, 2763‐2021, 2021; USDA, Foreign Agriculture ServiceChina MRLs, 2019). GB, 2763‐2021 covers the most common pesticides that are applied in the agricultural field and additionally covers chemical contaminants present in frequently consumed foods. China has also established regulations that observe MRL standards for primary manufacturing products, specifically for horticultural products such as fruit in various forms including juice beverages, dried and preserved fruits. GB, 2763‐2021 deals mainly with pesticide MRLs in fresh products; primarily in fruits, grains, vegetables, meat, eggs, and poultry.

The compliance of Australian farmers with pesticide applications is very high, which is demonstrated in the National Residue Survey (NRS) conducted every year. The samples tested covered over 6000 grain, fruit, and vegetable samples and the Department of Primary Industries and Regional Development, Agriculture and Food, Western Australia assists with investigations for residue detections over the MRL. The NRS survey for year 2020–2021 showed that there was 99.85% for animal food products and 99.17 % for plant products compliance with the MRLs surveyed (WA.gov.au, Department of Primary Industries and Regional Development, Agriculture and Food, 2021), and demonstrates a high level of residue compliance to pesticide MRLs within Australia. The Australian government runs an Imported Food Inspection Scheme to ensure imported foods are compliant with food standards (Australian government, DAWE, 1992). A report outlining pesticide breaches from foods imported into Australia from various countries (Table 2), found that foods from China failed to meet required levels on a total of 148 occasions between 2017 and 2019 (Amis, 2020). This compared to 93 instances from India, 44 from Vietnam, and 35 from Thailand.

**TABLE 2:** Countries pesticides breaching MRLs at AQIS (Amis, 2020)

Country	Number of pesticides breaches 2017–2019
China	148
India	93
Vietnam	44
Thailand	35

In 2009, data published by the FDA on the rejection of food consignments from China indicated food safety issues for seafood that include “unclean” product, product containing illegal additives, and noncompliance with respect to labeling and toxic residue limits (Gale & Buzby, 2009). These examples suggest that China needs to improve the control over chemical residue breaches in foods and implement an improved food safety management system to ensure conformance with regard to international food safety standards.

In Canada, the National Chemical Residue Monitoring Program (NCRMP) is complemented by the Food Safety Action Plan (FSAP). The sampling plans of the NCRMP focus on the analysis of registered products. These include the analysis of dairy, meat, poultry, horticultural products and medicines used to treat animals for chemical residues, pesticides/fungicides, irrigation water and soil, toxins originating from plants, mycotoxins, and heavy metals. The FSAP conducts studies focusing on emerging chemical hazards associated with particular types of foods or regional barriers in which the permissible MRLs are not as yet established. The findings from the investigations of the NCRMP and FSAP also play an important part in the risk management options.

In the United States, the setting of MRLs is conducted by the US Environmental Protection Agency. Guidance on how to assess MRLs can be sourced from e‐Code of Federal Regulations.

### 2.3 Microbiological risk assessment

FSANZ conducts food microbiological risk assessment by referring to the published scientific data. In order to ascertain the integrity of regulatory decisions, FSANZ utilizes the peer review process. The method that FSANZ has adopted by disseminating the information and seeking feedback from all the stakeholders to debate on the risk assessment through a consultative process ensures a thoroughly scrutinized risk assessment is achieved. In addition to national peer reviewers, FSANZ also seeks input from international experts in handling more complicated risk assessments (FSANZ, 2013b).

In China, the use of Quantitative Microbiological Risk Assessment (QMRA) has increased in the past two decades. Dong et al. (2015) concluded that in order to enhance the use of QMRA, an increase in planning and initiating QMRA through adoption in the decision‐making in food safety management and in developing the process controls in the manufacturing environment are needed

Contamination of food with pathogens is still regarded as the most critical food safety matter in China. China has been improving its risk management strategy by having the National Food Safety Standards (NFSSs) reviewing over the past decades the causative parameters that resulted in the contamination of food by pathogens that are often involved in foodborne outbreaks. When implementing microbial risk assessments, China will need to deal with a changing consumer diet preference, pathogens adaptation to new processing parameters, new and improved laboratory testing technologies, and new food safety standards (Chen et al., 2021). Chen et al. (2021) envisaged that a risk‐based strategy from microbiological risk assessment shall be more readily adopted than solely relying on finished product testing to ensure microbial food safety.

Both Canada and the United States use the Quantitative Microbial Risk Assessment (QMRA) method (Whelan et al., 2010) in conducting risk assessment for microbiological hazards. It takes into consideration the sensitivity evaluation approach and uncertainty and variability in risk assessment. Other factors include the estimation of the concentration of the pathogens present, microbial killing steps throughout the process that keep the pathogens under control, and exposure analysis. Dose–response estimation is widely used in the QMRA. In addition, QMRA also looks at the estimated health impact by examining the probability that illness could occur through exposure to the estimated concentration of pathogens involved.

### 2.4 Cohesiveness among all stakeholders including governmental and private entities in delivering food safety risk assessment

A high level of cohesiveness, communication, and transparency between stakeholders across the supply chain is important for the efficacy of a robust food safety risk assessment. For example, FSANZ operates as part of a food safety ecosystem that has the support of all the various stakeholders including government agencies, departments of health, hospitals, federal and state government research institutions, universities, food industries, food growers, and food service and retailers. This close holistic approach to collaboration among all the necessary entities allows the sharing of knowledge and this transparency leads to successful food safety risk assessments. In Canada, it is the Food Safety and Nutrition Quality Program that provides collaborative links with stakeholders both in governmental and private sectors. This leads to a cohesive network among the stakeholders, contributing to the delivering of an effective food safety risk‐assessment system. The governmental and private sectors in the United States are generally cohesive. However, the Food Safety Modernisation Act (FSMA) discovered there is a challenge involving tribal factors associated with the Indian American migrants who had settled in the United States and established themselves in the food business. Hence, the FSMA is working towards improving the inclusive behavior to take into consideration of language and cultural differences in integrating migrants into the education of the food safety awareness.

There is a gap in this area in the China food safety risk‐assessment system. Compared with FSANZ, Canada, and the United States, the evidence suggests that governmental and private sectors in China have yet to develop transparent and systematic collaboration, although it is recognized that they communicate closely with each other from time‐to‐time. It is suggested that China should improve its food safety risk‐assessment system by increasing collaboration and cohesion, and further increase transparency in the assessment process to enhance stakeholders' knowledge in this regard.

### 2.5 Capacity‐building in food safety risk assessment

A gap was identified in the capacity‐building capability associated with food safety risk assessment in China. This is because of the lack of food safety personnel with the right skills and practical experience in this area (Wu et al., 2018). In a survey conducted among ten developed and developing countries in which China was one of the participants, it was revealed that the lack of technical knowledge and infrastructure to conduct risk assessments was among the major impediments to the development of a risk‐based food safety analysis system (Food Safety Magazine, 2019). In order for a country to manage its food safety effectively through the development of a risk‐based food safety management system, all responsible agencies including agriculture, policy‐making government agency, department of health, business, food industry, and finance department of government must work together. In addition, food safety policy needs to be managed carefully to avoid conflict with other policies, such as the production sector in field‐based agriculture and other industrial entities. China could benefit from guidance by international countries, which have established an efficient system.

### 2.6 Resource investment in food safety management

The Australian and New Zealand governments regard food safety as an important area that warrants government priority, and consequently, prioritized finance and resources are allocated to manage food safety for the general population. In 2021, for the food and beverage sector, the Australian government announced it will invest AUD$1.3 billion on the modern manufacturing initiative, in which, food safety is one of the key areas that would receive attention. In 2014, the New Zealand government announced the intention of spending the budget surplus of NZ$86 million dollars on food safety investment. Similar attitudes prevail in the United States, Canada, and European member nations where the government contribution to food safety is high. In Canada, the government announced on January 22, 2021, that it will invest CAD$162.6 million to build up the management of its food safety agency, CFIA, over the next 5 years. In addition, the Canadian government is planning to spend CAD$40 million per year continuously to improve the efficiency of the food safety program to overcome pandemic disruptions that have damaged food business and export markets.

In the United States, the USDA announced on June 8, 2021, that the government has decided to invest more than US$4 billion to improve the food system following pandemic disruption. The funds aim to improve food production, bring big changes in food processing, provide efficient infrastructure for distribution, and reduce foodborne illness through food safety risk assessment and a rapid response management strategy. It is believed these activities will lower the burden on healthcare due to foodborne outbreaks and increase the life expectancy of the population (USDA, 2021).

In comparison, there is a gap in this area in China where there is lack of investment through finance or technical personnel resources. This led to the lack of qualified technical scientific personnel to conduct food safety risk assessments, which in turn has led to an inadequacy in the food safety management system (Wu & Chen, 2013). The insufficiently funded food safety management system has also led to ill‐equipped food safety inspectors to monitor food safety compliance in the manufacturing sector. China is therefore experiencing technology gaps (Wang & Rungsuriyawiboon, 2010) and there is a need for improvement in food safety capacity to align with other countries. China has taken steps to increase resources with the World Bank by applying for a loan of US$400 million and was granted the loan on March 25, 2021.

### 2.7 Scientific support for the food safety risk‐assessment system

In 2010, an incident occurred in the China where the media incorrectly reported *trans* fatty acids, which can be found in partially hydrogenated vegetable oils, as “poison at the table” (Liu et al., 2015) rather than presenting a moderate approach for the potentially detrimental effect of *trans* fatty acids on health outcomes (Oteng & Kersten, 2020). This resulted in a large number of people avoiding foods containing the *trans* fatty acids (National Expert Committee of Food Safety Risk Assessment, 2012). In Australia, any food safety claim must be substantiated by validated peer‐reviewed scientific proof; FSANZ implements measures to stop deceptive claims by establishing controls involving truthful and scientifically sound verification (FSANZ, 2007). With the emerging trend of social media, it provides an avenue for the China government to use it to provide a speedy response in rectifying any false unsubstantiated claims with regard to food safety concerns reported in the news.

In the United States, CFSAN conducts various risk assessments of illness related to foodborne pathogens and utilizes both science‐based quantitative and qualitative risk assessments to tackle food safety issues. One example of such a critical risk assessment is the study of the danger of contaminated horticultural products on farm, and the subsequent risk to public health. The United States has also published science‐based research on the risk of acquisition of Hep A virus from the consumption of contaminated fresh produce (Fiore, 2004), and another outlining the highly pathogenic avian influenza virus–infected birds, chicken, turkeys, other animals, and humans (FDA, 2019). In Canada, the CFIA employed a modeling tool known as the “Establishment‐based Risk Assessment for local food businesses” (ERA‐Food), which enables decisions to be made for resource allocation, depending on the food safety risks involved with a particular scenario. The scientific information collected from questionnaires from processing facilities is used to determine the frequency at which the processing facility warrants inspection.

### 2.8 A nationwide network in conducting food safety risk assessment

In Australia and New Zealand, the food control system is holistic and intergovernmental on a national level involving both federal and state governments. The main constituents of the system are establishment of policy, standards preparation, and oversight of implementation of the standards. The food policy is set by the Council of Australian Governments Legislation and Governance Forum on Food Regulation. Food legislation is governed by the Australian states and territories and local government councils (FSANZ Risk Analysis in Food Regulation, 2013c). In the United States, there is the nationwide network of food safety risk‐assessment system that encompasses the federal and state regulating bodies. The interconnected nationwide network is noted to enable the implementation of speedy corrective actions in food recall situations. In Canada, there is the nationwide network that includes the Safe Food for Canadian regulations and the Safe Food for Canadians Act, who work together to provide an improved streamlined system for food safety practices. The China food safety risk‐assessment system, on the other hand, appears to lack the cohesive integration of systems over the whole nation. It is anticipated that recent efforts of the Chinese National Centre for Food Safety Risk Assessment (CFSA) will continue to close these gaps, where they are working to develop a consolidated framework across China, and implement harmonized methodologies. Their actions are actively demonstrating a cooperative way of working together among all other central government sectors and should generate a sense of momentum for effective risk assessment outcomes.

### 2.9 Public trust in food safety systems

In Australia and New Zealand, any foods that are found to have potential hazards are effectively recalled by FSANZ, eliminating any further harm to public health. In the United States and Canada, generally, the public has full trust in the food safety systems governed by the governments (Food Safety Magazine, 2006 & Sutherland et al., 2020). However, with the nontraditional and novel food areas such as genetically modified (GM) food, there is a perception from a large proportion of the population that believe there is disagreement among the scientific professionals if GM foods are safe to consume (Pew Research Center, 2016). Conversely, a minority of the population has the perception that scientists are confident with the risks of GM foods on public health (Pew Research Center, 2016).

In the China food safety system, however, it is reported (Kendall et al., 2019) that consumers have lost confidence after witnessing numerous incidents that have involved food safety breaches happening in the food industry. This has led to a recent trend where Chinese nationals living in countries such as Australia act as personal shoppers, or “Daigou,” for consumable commodities manufactured locally to send back to customers back in China (Xiao & Mantesso, 2019). It clearly demonstrates Chinese consumers prefer imported foods over local product, particularly in chosen categories which they believe outperform those manufactured in China in both safety and quality. Since the 2008 milk scandal where Melamine was found in infant formula (Parry, 2008), China has put a lot of effort into tightening the laws and regulations regarding the dairy sector. Yet, consumers in China remain willing to seek out and pay premium prices for products imported from Australia and New Zealand that have a perceived greater guarantee of food safety and quality. The CFSA is looking to improve their food safety systems further, such that it builds trust for their consumers and provides assurance that products produced in China can be of similar safety and quality to other international products. It is predicted that the improvements in the China food safety system discussed earlier will assist the CFSA to build consumer trust.

### 2.10 Food fraud

As with the increase of international trade, there is an increasing level of food fraud in the global market. More often than not, food fraud is a result of the economic benefit a food manufacturer may yield through the illegal practice. The major aspect of food fraud focuses on the asymmetric information, food labeling, counterfeit foods, and food adulteration (Ehmke et al., 2019). Food fraud is not easy to detect, and often subtle differences in qualities can only be discovered via DNA molecular technology; hence, sellers are tempted to take the risk and engage in food fraud (Darby & Karni, 1973; Nelson, 1970). Inspection rates for food fraud by authorities are often low, and recurring offenders are not punished to a sufficient extent where repeat offending is deterred, leading to re‐offences. For example, advanced inspection departments such as the FDA of the United States still only inspects around 1% of imported foods.

One of the major food fraud issues facing Australian traders involves neighboring countries, including Asia and South‐East Asia countries, producing counterfeit products and labeling the products as produce of Australian origin. The Commonwealth Scientific and Industrial Research Organisation (CSIRO) is helping the government to find solutions to counteract food frauds that jeopardize products of Australian origin. One of the methods CSIRO is researching is to develop a national food provenance infrastructure to integrate data that can verify region and method of processing of a food product. CSIRO is working on generating isotopic fingerprints via the environmental markers from water and soil. These fingerprints are very specific to an agricultural region and can be used to verify claims about provenance, sustainability, and processing technologies. CSIRO is also developing real‐time autonomous sensor systems, such as the rapid evaporative ionisation mass spectrometry technology, which can be applied on agricultural farms and in food manufacturing sectors (CSIRO, 2021). Another widely committed food fraud centers around food labeling. Bimbo et al. (2019) developed a framework to investigate food labeling fraud that involves the collection of sales data, and the application of an empirical economic model. The framework was used in a case study to investigate the fraudulent “100 per cent Italian” claims on the Italian extra‐virgin olive oil market during the period 2014–2017, suggesting that the detrimental effect on the consumers on purchasing the counterfeit products far outweighed the gain by the producer of the counterfeit products. Moreover, they suggest an effective deterrent to food fraud may involve a loss in reputation for the company involved, rather than the imposing of bigger fines.

Honey fraud in the United States in 2001 has gained massive international attention. In 2001, an antidumping tariff was imposed on two countries implicated in the fraudulent export of honey, namely, Argentina and China. Here, the honey producers evaded tariffs via fraudulent labeling of origin. The most common ways noted to evade tariffs have been through mis‐manifesting and trans‐shipping (Ferrier, 2021). Mis‐manifesting refers to a trader falsely presenting to customs official a traded product upon importation that is different than its actual identity. Trans‐shipping, on the other hand, involves the intentionally covering up of an export product's true origin of region by illegally shipping the product via another country to reach the final destination. In these situations, there are various ways to detect fraud relating to place of origin. Data collected from trade transaction is certainly one of the most useful sources of information that can aid in origin fraud detection. The three methods employed by the US government as described by Ferrier (2021) in detecting origin fraud are trans‐shipping (the flows of the time‐linked trade transactions via a third country), excess trade (the amount of exports surpasses the amount of production), and gap in the import transaction (the discrepancies between the amount of origin‐reported exports and the amount of destination‐reported imports).

Global statistics indicate the food fraud incidences tend to be higher in China compared to other developed countries. In Australia, food fraud incidences are for the most part, limited and controlled. In Canada, food fraud prevention will be managed over the next 5 years by the Food Policy of Canada, which has invested $3.1 million for Health Canada to sponsor the CFIA work. This initiative led to the increase in staff members in 2019–2020 in handling food fraud matters. In the United States, US FSMA actively conducts reviews of food fraud activities to implement preventive actions to reduce food fraud incidences.

### 2.11 Preventing food safety breaches

FSANZ provides relevant processing guidelines that assist those in the food industry to prepare and implement Hazard Analysis Critical Control Point (HACCP) plans that ensure potential food safety breaches are prevented (Mortimore & Wallace, 2013). As part of such plans, in‐chain processing monitoring ensures the finished product is safe for consumption. In trying to develop a food safety culture, the Chinese government has rolled out a series of guidelines on the application of HACCP, such as GB/T 27342‐2009. However, the barriers to the broad and effective adoption of HACCP by the food industry include the limited opportunity for factory employees to upskill in these areas, as well as the cost for implementing and monitoring a HACCP plan and the lack of a surveillance program. As a result, many companies rely mostly on end‐product testing to assess food safety. Relying solely on the regular microbiological sampling of end‐products is not recommended for product quality or safety, and instead it is suggested that attention should be placed on the practice of good manufacturing practice and good hygienic practice, accompanied with a validated processing plan. It is of upmost importance that the close monitoring of critical control points as stipulated in the HACCP plan is the key element for controlling the microbial quality and safety of food (ICMSF, Microorganisms in Foods 8, 2011; Zwietering et al., 2016). Without the monitoring of intermediate products of the processing, the source of contamination cannot be identified solely relying on finished product testing.

### 2.12 Food handlers’ food safety knowledge

In Australia and New Zealand, FSANZ provides guidelines on food safety risks to all food regulators, which communicate the food safety requirements effectively to the food manufacturers and food handlers. This helps safeguard against serious food safety breaches that could be easily avoided (FSANZ, 2013a). However, in China, it is apparent there is a lack of hygienic food preparation knowledge and its ramifications by the food operators. For example, in 2019, a food poisoning incident was reported in the newspaper that involved students from a school near a factory which manufactured gluten strips coated with spices. The students were suffering severe gastrointestinal illness after consuming such products, and the cause of the food poisoning was determined to be microbial contamination due to the unhygienic conditions in which the gluten strips were manufactured (People's Daily Overseas New Media, 2019). Another reported case involves 36 students who were sent to hospital for treatment for gastrointestinal disease upon eating contaminated noodles made from mouldy potato starch (China Daily Newspaper, 2019). Food safety incidents such as these were caused by the lack of food hygiene and safety knowledge (Fan & Li, 2007). This appears to result from production managers prioritizing yield over food safety during production (Powell et al., 2011; Yiannas, 2009).

In the United States, the FDA implemented the FSMA, which requires farmers and food processors to comply with the food safety requirement. The FSMA consists of a few rules, namely, the Produce Safety rule, for Animal and Human food, there is the Preventive Controls rule and the Foreign Supplier Verification Programs rule. With food safety training considered a priority, the FDA collaborates with private as well as public sectors and federal agencies to deliver training to those who need it the most. Other collaborators include the food industry, international ties with global food safety organizations, and academia in fulfilling the training needs.

In Canada, the government also places high priority on the need for strong food safety education, which is provided by the Federal/Provincial/Territorial committee on Food Safety Policy (FPTCFSP). FPTCFSP's responsibility is to set up the food safety training requirements for food handlers and provide certification for recognizing training received. A summary of the comparison of food safety system of China, Australia/New Zealand, Canada, and the United States is presented in Table 3.

**TABLE 3:** A summary of the comparison of food safety systems among China and Australia/New Zealand, Canada, and the United States

Activities	China	Australia/New Zealand	Canada	USA
Risk Assessment Methodologies	As per Codex Alimentarius	As per Codex Alimentarius	As per Codex Alimentarius	As per Codex Alimentarius
Chemical risk assessment	Pesticide MRLs are developed mainly in fresh foods, primarily in vegetables, fruits, grains, meat, eggs, and poultry with limited coverage of processed food	Pesticide MRLs are developed for primary fresh produce and processed foods	“Ranked Risk Assessment” system;	FDA‐iRISK^®^—web‐based comparative chemical risk‐assessment system
			‘Risk model scoring system’; Risk Priority Compound List	
Microbiological risk assessment	Quantitative microbial risk assessment system	Quantitative microbial risk assessment system	Quantitative microbial risk assessment system	Quantitative microbial risk assessment system
Nationwide risk assessment	Limited coordinated framework across China	Multijurisdictional activities among central government ministries and state‐wide regulators	Governmental health agencies, non‐governmental institutions, academia, research institutions all work within Health Canada to collaborate together	FDA and CFSAN manage the nationwide food safety risk assessment
Cohesiveness among stakeholders	Cohesiveness among governmental departments and private industry is lacking	Close collaboration among scientific research sectors, academic institutes, governmental agencies and private industry	Food Safety and Nutrition Quality Program provides collaborative links with stakeholders providing a cohesive network	Overall, there is cohesiveness among stakeholders, Food Safety Modernisation Act rules face tribal (Indian American) challenge
Resource investment	In 2021, the World Bank granted China a loan of US$400 million for the improvement projects on food safety	The Australian government announced in 2021 investing AUD$1.3 billion on modern manufacturing initiative in which food safety will receive a big allocation. New Zealand government announced in 2014 aiming to spend the budget surplus of NZ$86 million on food safety.	The Canadian government announced in 2021 that it will invest CAD$162.6 million for management of the food safety agency	United States Department of Agriculture (USDA) announced in 2021 that government will invest more than US$4 billion to improve the food system
Food Fraud	Global statistics indicate the food Fraud incidences tend to be high compared to other developed countries	Food Fraud incidences are limited and controlled	Food Fraud prevention is managed over next 5 years by Food Policy of Canada which invests $3.1 million for Health Canada to sponsor the Canadian Food Inspection Agency (CFIA) work. This initiative led to the increase in staff members in 2019–2020 in handling food fraud matter.	US Food Safety Modernisation Act actively conducts reviews of Food Fraud activities to implement preventive actions to reduce food fraud incidences.
Effort to prevent food safety breaches	China government rolled out GB/T 27,342 guideline to HACCP, which is a step forward to enhance food safety breaches prevention.	Concerted effort to ensure food handlers adhere to regulation, all food must be manufactured under a food safety plan including a which includes HACCP and requires approval by state government regulators in Australia and in New Zealand, it is governed by New Zealand Food Safety	Canadian Institute of Food Safety (CIFS): provision of Food Handlers Certification and HACCP program to food manufacturers and food handlers	Federal Regulatory Programs: FDA, USDA, EPA, CFSAN.
				State and Local Regulatory System: DHHS.
				HACCP system.
Food handlers’ food safety knowledge	Limited knowledge by food handlers is causing food safety breaches that could have been substantially prevented. Full coverage of regular auditing of manufacturing industry is made difficult by the large number of industry players involved	Food safety training well disseminated and regulated in food manufacturing industry and regulators conduct auditing of food manufacturers regularly to ensure compliance	CIFS provide Certification of Food Handler and HACCP plan training	FDA provides various training tools for food handlers in ensuring proper hygiene practice is adhere to by food handlers and manufacturers

*Source*: Table prepared from AGAPVMA (2020); Bietlot and Kolakowski (2012); CFIA (2021); CNCFSA (2021); Gale and Buzby (2009); FSANZ (2013a); GB 2763‐2019 (2019); Health Canada (2021); ICMSF (2011); Li and Liu (2017); USDA (2021); Wallace and Oria (2010); Wu et al. (2018); Wu and Chen (2013).

## 3 DISCUSSION

In terms of pesticide residue MRLs, China has established extensive limits that industry must adhere to for fresh food, primarily in fresh vegetables, fruits, eggs and meat; however, limited coverage remains for processed food. In contrast, pesticide residue MRLs are well established both in fresh and processed food in Australia, New Zealand, the USA and Canada. Canada employs the more sophisticated “Ranked Risk Assessment” system and likewise, USA uses the advanced iRISK computer software to assist in the pesticide residue assessment. For microbiological risk assessment, China, Australia, New Zealand, the United States, and Canada use the world‐renowned Quantitative Microbiological Risk‐Assessment system.

As for government investment in food safety, China applied for a loan from the World Bank of $400 million in 2021 for funding to improve food safety. The loan was approved, thereby providing resources to further improve food safety (Food Safety Magazine, 2021). Other countries have also invested varying amounts of money in raising their food safety standards. Australian government invested AUD$1.3 billion in 2021 in modern manufacturing where food safety forms an integral part of the initiative. A NZ$86 million food safety program was announced by the New Zealand government in 2014. The Canadian government invested Can$162.6 million in food safety in 2021. In the United States, the USDA announced in 2021 that it would invest more than USD$4 billion to improve food safety. When it comes to food fraud, global statistics indicate that China has a higher incidence compared to other countries. The Canadian government invested Can$3.1 million to sponsor Canadian Food Inspection Agency to hire more staff members to handle food fraud cases in 2019–2020. To reduce the prevalence of food fraud, the FSMA is effective in the United States. As part of the efforts to improve food safety, China has implemented HACCP practice in the manufacturing sector, which is consistent with the practices used by other developed countries including Australia, New Zealand, Canada, and the United States. With regard to the food handlers' food safety knowledge, China is expanding its education policy on food handlers with the focus on how poor food handling can lead to lethal consequences for public health. In Australia, New Zealand, Canada, and the United States, food handlers are very often required to possess an accredited qualification for food handling. This can be used as a guide to develop food handlers' food safety knowledge in China.

## 4 CONCLUSION

The goal of the review is to compare the food safety risk‐assessment systems utilized by China to that of Australia/New Zealand, Canada, and the United States. As global trade is crucial to the prosperity of any country's economy, it is obvious that it is beneficial for the exporting and importing countries to agree on food safety standards. Due to China's vast amount of export and import trade with other countries, China is actively improving a food safety system that aligns with the Codex Alimentarius Commission and is in accordance with other developed countries' food safety standards.

China is demonstrating promising potential in attaining the same level of food safety risk‐assessment systems as per other developed countries, demonstrated through the clear government prioritization to engage even further with the Codex Alimentarium and other international experts to achieve the best standard in food safety. China has improved its food safety system with active involvement in Codex committees with various aspects of food safety risk assessment and has contributed to international chemical and microbiological risk assessments. There are still areas of improvement that can enhance China food safety system, which include reducing food fraud incidence and increasing food safety education for food handlers among others. In seeking a loan from the World Bank to increase its investment in food safety, China demonstrated its commitment to increasing food safety measures.

China has been actively engaged in a lot of different proposals initiated by the *Codex Committee* on Food Additives (CCFA) since 1984, toxicology data provided by China helps to cement a better understanding of food additives impact on human health and raise more knowledge in the scientific community on the danger of toxic and carcinogenic effect of food additives to human health (Liu et al., 2019).

The creation of CFSA in 2009 has since also brought a lot of positive changes to strengthen food safety risk assessment in China. However, due to the vast number of manufacturing industries involved and what is assumed to be the inherent attitudes of people with financial gain as the key motivating factor, effort is still needed to further improve the food safety risk‐assessment systems compared to other countries. By enhancing the role and function of CFSA, China will undoubtedly attain an improved and effective food safety risk‐assessment system.

## AUTHOR CONTRIBUTIONS

**Sieh Ng**: Conceptualization; Methodology; Project administration; Resources; Writing – original draft; Writing – review & editing. **Shuyan Shao**: Conceptualization; Methodology; Project administration; Resources; Writing – original draft; Writing – review & editing. **Nan Ling**: Conceptualization; Funding acquisition; Project administration; Resources; Writing – original draft; Writing – review & editing

## CONFLICT OF INTEREST

There are none to declare

## References

[jfds16334-bib-0001] Agriculture law of the people's republic of China . (2003). https://wipolex‐reswipo.int/edocs/lexdocs/laws/en/cn/cn157en.pdf

[jfds16334-bib-0002] Amis, A. (2020). Pesticide breaches and Australian imported food 2017–2019 . (https://www.pesticides.australianmap.net)

[jfds16334-bib-0003] Archer, N. , Krause, D. , & Logan, A. (2017). Personalised food revolution. Food Australia, July/August, 42–43.

[jfds16334-bib-0004] Askew, K. (2021). *Food Navigator.com newsletter*, 29 Jan 2021.

[jfds16334-bib-0005] Australian Government Australian Pesticides Veterinary Medicines Authority (2020). Chemicals and Products: Acceptable daily intakes for agricultural and veterinary chemicals.

[jfds16334-bib-0006] Australian Government DAWE . (1992). Imported Food Inspection Scheme. https://www.awe.gov.au/biosecurity‐trade/import/goods/food/inspection‐compliance/inspection‐scheme

[jfds16334-bib-0007] Betts, J. A. , & Gonzalez, J. T. (2016). Personalised nutrition: What makes you so special? British Foundation Nutrition Bulletin, 41, 353–359.

[jfds16334-bib-0008] Bietlot, H. , & Kolakowski, B. (2012). Risk assessment and risk management at the Canadian Food Inspection Agency (CFIA): A perspective on the monitoring of foods for chemical residues. Drug Testing and Analysis, 4(1), 50–58. 22851361 10.1002/dta.1352

[jfds16334-bib-0009] Bimbo, F. , Bonanno, A. , & Viscecchia, R. (2019). An empirical framework to study food labelling fraud: an application to the Italian extra‐virgin olive oil market. Australian Journal of Agricultural and Resource Economics, 63(4), 701–725.

[jfds16334-bib-0010] Buckow, R. , Ng, S. , & Toepfl, S. (2013). Pulsed electric field processing of orange juice: A review on microbial, enzymatic, nutritional, and sensory quality and stability. Comprehensive Reviews in Food Science and Food Safety, 12, 455–467. 33412665 10.1111/1541-4337.12026

[jfds16334-bib-0011] Canadian Food Inspection Agency . (2021). https://inspection.canada.ca/eng/1297964599443/1297965645317

[jfds16334-bib-0012] Chadwick, J. (2017). Here's how 3D food printers are changing what we eat. *Tech Republic*. https://www.techrepublic.com/article/heres‐how‐3d‐food‐printers‐are‐changing‐the‐way‐we‐cook/

[jfds16334-bib-0013] Chen, X. , Lyu, H. , Zhang, J. , Bai, L. , & Wang, J. (2021). National food safety standards related to microbiological contaminants in china: recent progress and challenges. Foodborne Pathogens and Disease, 18(8), 528–537. 10.1089/fpd.2021.0022

[jfds16334-bib-0014] Chen, Z. , Dong, F. , Xu, J. , Liu, X. , & Zheng, Y. (2015). Management of pesticide residues in China, Journal of Integrative Agriculture, 14(11), 2319–2327.

[jfds16334-bib-0015] China Daily Newspaper . (18 March 2019). Safety of food at schools scrutinized. https://www.chinadaily.com.cn/a/201903/18/WS5c8ed314a3106c65c34ef0d5.html

[jfds16334-bib-0016] China National Centre for Food Safety Risk Assessment Department of Primary Industries and Regional Development , Agriculture and food . (2021). https://www.agric.wa.gov.au/pests‐weeds‐diseases/control‐methods/chemicals/preventing‐residues/residues‐crops

[jfds16334-bib-0017] CSIRO – Verifying food credential (2021). https://www.csiro.au/en/about/challenges‐missions/Trusted‐Agrifood‐Exports/Building‐an‐Australian‐food‐provenance‐infrastructure

[jfds16334-bib-0018] Darby, M. , & Karni, E. (1973). Free competition and the optimal amount of fraud, Journal of Law and Economics, 16, 67–88.

[jfds16334-bib-0019] Dong, Q. L. , Barker, G. C. , Gorris, L. G. M. , Tian, M. S. , Song, X. Y. , & Malakar, P. K. (2015). Status and future of quantitative microbiological risk assessment in China. Trends in Food Science and Technology, 42(1), 70–80. 26089594 10.1016/j.tifs.2014.12.003 PMC4460287

[jfds16334-bib-0020] Ehmke, M. D. , Bonanno, A. , Boys, K. , & Smith, T. G. (2019). Food fraud: economic insights into the dark side of incentives. Australian Journal of Agricultural and Resource Economics, 63(4), 685–700.

[jfds16334-bib-0021] Ensuring Safe Food: From Production to Consumption (1998). Institute of Medicine (US) and National Research Council (US) Committee to Ensure Safe Food from Production to Consumption. National Academies Press (US). 24967491

[jfds16334-bib-0022] Fan, Y. , & Li, X. (2007). Status and challenge of food hygienic standard system in China. Chinese Journal of Food Hygiene, 19(6), 505–508.

[jfds16334-bib-0023] FDA (2019). Diagnostic Targets and Potential Vaccine Against H5N1 Avian Influenza . https://www.fda.gov/science-research/licensing-and-collaboration-opportunities/diagnostic-targets-and-potential-vaccine-against-h5n1-avian-influenza

[jfds16334-bib-0024] FDA iRISK (2021). https://irisk.foodrisk.org/

[jfds16334-bib-0025] Ferrier, P. M. (2021). Detecting origin fraud with trade data: the case of U.S. honey imports. Australian Journal of Agricultural and Resource Economics, 65(1), 222–245.

[jfds16334-bib-0026] Fiore, A. (2004). Hepatitis A transmitted by food. Food Safety, 2004, 38. 10.1086/381671 14986256

[jfds16334-bib-0027] Fischer, R. H. , De Jong, A. E. I. , De Jonge, R. , Frewer, L. J. , & Nauta, M. J. (2005). Improving food safety in the domestic environment: the need for a transdisciplinary approach. Risk Analysis, 25, 503–517. 16022686 10.1111/j.1539-6924.2005.00618.x

[jfds16334-bib-0028] Food Safety Magazine , June 1, 2006. Shopping for Food Safety and the Public Trust: What Supply Chain Stakeholders Need to Know About Consumer Attitudes. https://www.food‐safety.com

[jfds16334-bib-0029] Food Safety Magazine , March 29, 2021. World Bank Approves $400 Million Loan for China for Food Safety Management. https://www.food‐safety.com

[jfds16334-bib-0075] Food Safety Magazine EDIGEST , July 2, 2019. Improving Capacity‐Building for Food Safety Risk Assessment in Developing Countries.

[jfds16334-bib-0030] FoodRisk.org (2017). FoodRisk.org is a metadatabase of tools and models for food safety professionals in industry, academia, and government. https://www.foodrisk.org/

[jfds16334-bib-0032] FSANZ . (2007). “Nutrition, Health and related Claims” A guide to the development of a food standard for Australia and New Zealand . https://www.foodstandards.gov.au/industry/labelling/pages/nutrition‐health‐and‐related‐claims.aspx

[jfds16334-bib-0033] FSANZ . (2013a). Communicating food‐related health risks . https://www.foodstandards.gov.au/publications/riskanalysisfoodregulation/Documents/risk-analysis-food-regulation-ch7-pdf.pdf

[jfds16334-bib-0034] FSANZ . (2013b). Managing food‐related health risks . https://www.foodstandards.gov.au/publications/riskanalysisfoodregulation/Documents/risk-analysis-food-regulation-ch6-pdf.pdf

[jfds16334-bib-0035] FSANZ . (2013c). Risk analysis in food regulation . https://www.foodstandards.gov.au/publications/riskanalysisfoodregulation/Pages/default.aspx

[jfds16334-bib-0036] Gale, F. , & Buzby, J. C. (2009). Import from China and Safety Issues. USDA Economic Information Bulletin Number, 52, July 2009.

[jfds16334-bib-0037] GB 2763‐2021 . (2021). *National Food Safety Standard—Maximum residue limits for pesticides in food*. National Health and Family Planning Commission of the People's Republic of China and Ministry of Agriculture of the People's Republic of China.

[jfds16334-bib-0076] GB 2763‐2019 . (2019). *National food safety Standard*—Maximum residue limits for pesticides in food. National Health and Family Planning Commission of the People's Republic of China and Ministry of Agriculture of the People's Republic of China.

[jfds16334-bib-0176] GB/T 27342‐2009 . (2009). *National Food Safety Standard*—Hazard analysis and critical control point (HACCP) system—Requirements for dairy processing plant. National standards of People's Republic of China. www.ChineseStandard.net

[jfds16334-bib-0038] Health Canada . (2021). Food and Drugs Act (R. S. C., 1985, c. F‐27) https://www.canada.ca/en/health‐canada.html

[jfds16334-bib-0039] International Commission On Microbiological Specifications for Foods (ICMSF) Microorganisms in Foods 8 . Use of data for assessing process control and product acceptance . (2011). Springer link.

[jfds16334-bib-0040] Jackson, L. A. , & Jansen, M. (2010). Risk assessment in the international food safety policy arena. Can the multilateral institutions encourage unbiased outcomes? Food Policy, 35, 538–547.

[jfds16334-bib-0041] JECFA . (2021). Chemical and technical assessments of food additives (CTAs). https://www.who.int/foodsafety/areas_work/chemical‐risks/jecfa/en/

[jfds16334-bib-0042] JEMRA . (2021). https://www.fao.org/food/food‐safety‐quality/scientific‐advice/jemra/en/

[jfds16334-bib-0043] Juliano, P. , Knoerzer, K. , Sellahewa, J. , Nguyen, M. , & Buckow, R. (2021). Food engineering innovations across the food supply chain (1st ed). Academic Press.

[jfds16334-bib-0044] Kendall, H. , Kuznesof, S. , Dean, M. , Chan, M ‐ Y. , Clark, B. , Home, R. , Stolz, H. , Zhong, Q. , Liu, C. , Brereton, P. , & Frewer, L. (2019). Chinese consumer's attitudes, perceptions and behavioural responses towards food fraud. Food Control, 95, 339–351.

[jfds16334-bib-0045] Knoerzer, K. , Juliano, P. , Roupas, P. , & Versteeg, C. (2011). Innovative food processing technologies: Advances in multiphysics simulation. John Wiley & Sons, Ltd. and Institute of Food Technologists.

[jfds16334-bib-0046] Knoerzer, K. , & Muthukumarappan, K. (2021). Innovative food processing technologies (1st ed.), Elsevier.

[jfds16334-bib-0047] Lanscape News . (2020). How fermentation's new heyday is benefitting human and planetary health. 29 April 2020. https://news.globallandscapesforum.org/44165/how‐fermentations‐new‐heyday‐is‐benefitting‐human‐and‐planetary‐health/

[jfds16334-bib-0048] Li, N. , & Liu, Z. (2017). Risk assessment in China: capacity building and practices. Food Safety in China, 271–285.

[jfds16334-bib-0049] Liu, Z. , Liu, B. , & Chen, G. (2019). Retrospective analysis of the development history of the Chinese food additive standards system based on the CODEX principles. NPJ Science of Food, Dec16, 3(27), 1–6. 10.1038/s41538-019-0060-x 31872064 PMC6914770

[jfds16334-bib-0050] Liu, Z. P. , Zhou, P. P. , Mao, W. F. , & Li, N. (2015). Trans fatty acid levels in foods and intakes among population aged 3 years and above in Beijing and Guangzhou cities, China. Biomedical and Environmental Sciences, 28(7), 477–485. 26248731 10.3967/bes2015.069

[jfds16334-bib-0051] Ministry for Primary Industries, New Zealand Government (2021). https://www.mpi.govt.nz/agriculture/agricultural‐compounds‐vet‐medicines/maximum‐residue‐levels‐agricultural‐compounds/

[jfds16334-bib-0052] Mortimore, S. , & Wallace, C. (2013). HACCP—A Practical Approach (3rd ed). Springer.

[jfds16334-bib-0053] National Expert Committee of Food Safety Risk Assessment . (2012). Risk assessment of dietary intake of trans fatty acids in Chinese population . https://www.cfsa.net.cn:8033/UpLoadFiles/news/upload/2013/2013‐11/0ea2121b‐1ac3‐475a‐b77c‐e97342046b71.pdf

[jfds16334-bib-0054] Nelson, P. (1970). Information and consumer behaviour. Journal of Political Economy, 78, 311–329.

[jfds16334-bib-0055] Oteng, A. B. , & Kersten, S. (2020). Mechanisms of action of trans fatty acids. Advances in Nutrition, 11(3), 697–708. 31782488 10.1093/advances/nmz125 PMC7231579

[jfds16334-bib-0056] Parry, J. (2008). China's tainted infant formula sickens nearly 13 000 babies. British Medical Journal, 337. doi: 10.1136/bmj.a1802 18815175

[jfds16334-bib-0057] People's Daily Overseas New Media . March 26, 2019. Latiao faces sales ban near schools over health concerns.

[jfds16334-bib-0058] Pew Research Center , Dec 1, 2016. The new food fights: U. S. public divides over food science . www.pewresearch.org

[jfds16334-bib-0059] Powell, D. A. , Jacob, C. J. , & Chapman, B. J. (2011). Enhancing food safety culture to reduce rates of foodborne illness. Food Control, 22(6), 817–822. 10.1016/j.foodcont.2010.12.009

[jfds16334-bib-0060] Sutherland, C. , Sim, C. , Gleim, S. , & Smyth, S. J. (2020). Consumer insights on Canada's food safety and food risk assessment system. Journal of Agriculture and Food Research, 2, 1–9.

[jfds16334-bib-0061] Tam, W. , & Yang, D. (2005). Food Safety and the development of regulatory institutions in China. Asian Perspective, 29(4), 5–36.

[jfds16334-bib-0078] USDA, Foreign Agriculture Service, US Department of Agriculture . (2019). Data and Analysis. Releases Standard for Maximum Residue Limits in Foods, June 4, 2019. https://www.fas.usda.gov/data/china-national-food-safety-standard-maximum-residue-limits-pesticides-foods

[jfds16334-bib-0062] USDA . (2021). USDA to Invest More Than $4 Billion to Strengthen Food System. https://www.usda.gov/media/press-releases/2021/06/08/usda-invest-more-4-billion-strengthen-food-system

[jfds16334-bib-0063] Wallace, R. B. , & Oria, M. (2010). *Enhancing food safety: The role of the food and drug administration* . National Research Council (US) Committee on the Review of Food and Drug Administration's Role in Ensuring Safe Food. National Academies Press (US). 25032388

[jfds16334-bib-0064] Wang, X. , & Rungsuriyawiboon, S. (2010). Agricultural efficiency, technical change and productivity in China. Post‐Communist Economies, 22(2), 207–227.

[jfds16334-bib-0077] Wang, S. P. (2010). Food standards and laws. Beijing: Science Press.

[jfds16334-bib-0080] WA.gov.au. Department of Primary Industries and Regional Development, Agriculture and Food. (2021).

[jfds16334-bib-0065] Watkins, P. , Logan, A. , & Bhandari, B. (2022). Three‐dimensional (3D) food printing—An overview. In Juliano, J. , Knoerzer, K. , Sellahewa, J. , Nguyen, M. , & Buckow, R. (Ed.). Food engineering across the supply chain. Elsevier. 261–270.

[jfds16334-bib-0066] Whelan, G. , Tryby, M. E. , Pelton, M. A. , Soller, J. A. , & Castleton, K. J. (2010). Using an integrated, multi‐disciplinary framework to support quantitative microbial risk assessment. International Congress on Environmental Modelling and Software, 93.

[jfds16334-bib-0067] Wu, Y. , & Chen, Y. (2013). Food safety in China. Journal of Epidemiology and Community Health, 67(6), 478–479. 23322847 10.1136/jech-2012-201767

[jfds16334-bib-0068] Wu, Y. , Liu, P. , & Chen, J. (2018). Food safety risk assessment in China: Past, present and future. Food Control, 90, 212–221.

[jfds16334-bib-0069] Xiao, B. , & Mantesso, S. (2019). The daigou channel—how a handful of Chinese shoppers turned into a billion‐dollar industry. Australia ABC NEWS, 31 Jul 2019.

[jfds16334-bib-0070] Yiannas, F. (2009). Food safety culture: Creating a behavior‐based food safety management system. Springer Science. 10.1007/978-0-387-72867-4

[jfds16334-bib-0071] Yuan, Z. , Wan, G. , & Khor, N. (2011). The rise of the middle class in the People's Republic of China. ADB Economics Working Paper Series, *247*.

[jfds16334-bib-0072] Zhang, Z. , Godefroy, S. B. , Lyu, H. , Sun, B. , & Fan, Y. (2018). Transformation of China's food safety standard setting system—Review of 50 years of change, opportunities and challenges ahead”. Food Control, 93, 106–111.

[jfds16334-bib-0073] Zwietering, M. H. , Jacxsens, L. , Membré, J. M. , Nauta, M. , & Peterz, M. (2016). Relevance of microbial finished product testing in food safety management. Food Control, 60, 31–43.

